# Acute care utilization due to hospitalizations for pediatric lower respiratory tract infections in British Columbia, Canada

**DOI:** 10.1186/1472-6963-12-451

**Published:** 2012-12-08

**Authors:** Pablo Santibanez, Katherine Gooch, Pamela Vo, Michelle Lorimer, Yurik Sandino

**Affiliations:** 1 Sauder School of Business, University of British Columbia, 2053 Main Mall, Vancouver, BC, V67 1Z2, Canada; 2 Abbott Laboratories, 200 Abbott Park Road, Abbott Park, IL, 60064, USA; 3 Lorimer Enterprises, Inc., 104 Dixon Crescent Red Deer, Alberta, T4R 2H5, Canada; 4 Fraser Health Authority, Suite 400, Central City Tower 13450-102nd, Avenue Surrey, BC, V37 0H1, Canada

**Keywords:** Pediatrics, Lower respiratory tract infection, Acute care resource utilization, British Columbia

## Abstract

**Background:**

Pediatric LRTI hospitalizations are a significant burden on patients, families, and healthcare systems. This study determined the burden of pediatric LRTIs on hospital settings in British Columbia and the benefits of prevention strategies as they relate to healthcare resource demand.

**Methods:**

LRTI inpatient episodes for patients <19 years of age during 2008–2010 were extracted from the BC Discharge Abstract Database. The annual number of acute care beds required to treat pediatric LRTIs was estimated. Sub-analyses determined the burden due to infants <1 year of age and high-risk infants. Population projections were used to forecast LRTI hospitalizations and the effectiveness of public health initiatives to reduce the incidence of LRTIs to 2020 and 2030.

**Results:**

During 2008–2010, LRTI as the primary diagnosis accounted for 32.0 and 75.9% hospitalizations for diseases of the respiratory system in children <19 years of age and infants <1 year of age, respectively. Infants <1 year of age accounted for 47 and 77% hospitalizations due to pediatric LRTIs and pediatric LRTI hospitalizations specifically due to respiratory syncytial virus (RSV), respectively. The average length of stay was 3.1 days for otherwise healthy infants <1 year of age and 9.1 days for high-risk infants (P <0.0001). 73.1% pediatric LRTI hospitalizations occurred between November and April. Over the study timeframe, 19.6 acute care beds were required on average to care for pediatric LRTIs which increased to 64.0 beds at the peak of LRTI hospitalizations. Increases in LRTI bed-days of 5.5 and 16.2% among <19 year olds by 2020 and 2030, respectively, were predicted. Implementation of appropriate prevention strategies could cause 307 and 338 less LRTI hospitalizations in <19 year olds in 2020 and 2030, respectively.

**Conclusion:**

Pediatric LRTI hospitalizations require significant use of acute care infrastructure particularly between November and April. Population projections show the burden may increase in the next 20 years, but implementation of effective public health prevention strategies may contribute to reducing the acute care demand and to supporting efforts for overall pediatric healthcare sustainability.

## Background

Lower respiratory tract infections (LRTIs) in children and infants are common and can require hospitalization; thus, they are a major public health concern. A number of viruses including respiratory syncytial virus (RSV), influenza viruses, parainfluenza viruses, rhinovirus, and adenovirus are responsible for LRTIs. In children and infants, RSV is the most common cause; during the first year of life 50%–70% of children will have an RSV infection, and by the third year of age almost all children will have been infected.^1^

Neonatal and pediatric LRTIs and RSV are often mild, but can be associated with significant health resource utilization and result in an important burden on the patients, families, infrastructure, and budget of public healthcare systems [1]. Economic analyses indicate that substantial costs are associated with outpatient physician office and emergency department visits, but that a majority of the financial burden is accounted for by costs in inpatient hospital settings [2,3]. LRTI and RSV/LRTI hospitalization rates for all children <2 years of age vary between 0.5% and 2% [4]. In comparison, LRTI, and particularly RSV specific LRTI, hospitalization rates and lengths of stay in infants and high-risk infants are substantially increased and associated with additional costs [3,4]. High-risk infants include early and late-preterm infants and those with bronchopulmonary dysplasia and congenital heart disease [3,5].

In Canada, the annual cost of RSV-associated illness is over $18 million, and inpatient services associated with RSV/LRTI hospitalizations account for 62% of these annual expenditures [2]. Over the past two decades, the impact of LRTI hospitalizations on Canadian health resource utilization has increased as the incidences of admissions have risen from approximately 6,200 to 12,000 hospitalizations per year in children <2 years of age [2,6,7]. In addition, there is a pronounced seasonal variation in the incidence of LRTIs. This is likely due to the RSV season which is defined by the timing of the onset and offset of increased RSV circulation in a community. In northern hemisphere countries, the RSV season usually runs from December through April and is associated with significantly increased pediatric admissions for LRTIs [8].

The burden of disease associated with LRTIs may be reduced by effective public health prevention strategies that decrease hospitalizations and reduce lengths of stay [4]. Indeed, epidemics such as the most recent H1N1 outbreak led to rapid and important health prevention and public health initiatives to try to reduce the risk of transmission and health morbidity [9,10]. Although the effectiveness of such public health awareness campaigns are difficult to ascertain, any strategies that can decrease the incidence of severe viral respiratory infections are expected to have a considerable and important impact on acute care settings.

To date there is limited information describing the burden of pediatric LRTIs and the impact of public health prevention strategies directed against the infections causing LRTIs on hospital settings. In large countries such as Canada, reducing the present and projected burden of LRTIs and RSV on the public healthcare system involves detailed evaluations of the impact of these diseases on acute care in defined regions. In Canada, it is likely that identification of the burden of a disease in a specific province will enable the development of effective public health initiatives to reduce the incidence of the disease in this province. The experience in one province may serve as a model for provinces and territories throughout the rest of the country. Accordingly, the goal of these analyses was to determine the burden of pediatric LRTIs on hospital settings in British Columbia and to identify the benefits of prevention strategies as they relate to healthcare resource demand.

## Methods

The objectives of the study were to describe the overall burden and inpatient resources required to care for pediatric LRTI hospitalizations in British Columbia from April 2008 to March 2010. The patient and hospital burden as it relates to the length of stay in hospital and the differences between pediatric sub populations including those <1 year of age and those defined as high-risk were determined. Population estimates in British Columbia for 2008–2010 and projections to 2020 and 2030 were used to model the projected demand for pediatric LRTI hospitalizations up to 2020 and 2030. In addition, quantitative estimates of the effects of non-pharmacological interventions to decrease the incidence of LRTIs were generated to assess how the impact of effective public health prevention strategies relate to decreased demand on hospital settings in this province.

The pediatric population was defined as children younger than 19 years of age at the time of hospital admission [11,12]. Pediatric population estimates for 2008–2010 and growth statistics were obtained from BC STATS [13]. BC STATS is the official source for population figures within British Columbia health authorities. The analyses were based on the PEOPLE 35 [13] release which contains population counts by year, gender, local health area, and individual year of age. Data projecting pediatric population growth for 2020 and 2030 were obtained from the BC STATS component/cohort-survival model [13].

Hospital discharge records with an International Classification of Diseases, Tenth Revision (ICD-10) code identifiable as LRTI as primary diagnosis during the 2008–2009 (n=2498) and 2009–2010 (n=2090) fiscal years (April 1^st^ to March 31^st^) for inpatient separations associated to pediatric patients from any acute care hospital in British Columbia were obtained from the Canadian Institute for Health Information (CIHI) Discharge Abstract Database (DAD) [14]. Codes to identify an admission due to an LRTI are shown in Table 1[15]. The DAD contains data on facility discharges across Canada including demographic, administrative, and clinical data for hospital discharges (inpatient acute, chronic, and rehabilitation) and day surgery interventions. Co-morbidities and other conditions were derived from ICD-10 diagnosis codes specified in any of the first 10 diagnosis fields in the DAD published population. Data for this project was provided by the Fraser Health Authority (FHA). Access to the data was approved by FHA’s research office. An ethics approval exemption was granted on the basis of the study using unidentifiable non-clinical/administrative data at an aggregate level for purposes of reviewing workload and resource utilization and planning capacity into the future.

**Table 1:** International Classification of Diseases, Tenth Revision (ICD-10) codes identifying for lower respiratory tract infections

**ICD-10 Code**	**Description**
J10.1	Influenza due to other influenza virus with
J10.81	Influenza gastroenteritis
J10.89	Influenza due to other influenza virus with
J12.0	Adenoviral pneumonia
J12.1	Respiratory syncytial virus pneumonia
J12.2	Parainfluenza virus pneumonia
J12.3	Pneumonia due to SARS-associated coronavirus
J12.8	Other viral pneumonia
J12.81	Pneumonia due to SARS-associated coronavirus
J12.89	Other viral pneumonia
J12.9	Viral pneumonia, unspecified
J13.0	Pneumonia due to Streptococcus pneumoniae
J14.0	Pneumonia due to Hemophilus influenzae
J15.0	Pneumonia due to Klebsiella pneumoniae
J15.1	Pneumonia due to Pseudomonas
J15.20	Pneumonia due to staphylococcus, unspecified
J15.21	Pneumonia due to Staphylococcus aureus
J15.29	Pneumonia due to other staphylococcus
J15.3	Pneumonia due to streptococcus, group B
J15.4	Pneumonia due to other streptococci
J15.5	Pneumonia due to Escherichia coli
J15.6	Pneumonia due to other aerobic Gram-negative bacteria
J15.7	Pneumonia due to Mycoplasma pneumoniae
J15.8	Pneumonia due to other specified bacteria
J15.9	Unspecified bacterial pneumonia
J16.0	Chlamydial pneumonia
J16.8	Pneumonia due to other specified infectious organisms
J17.0	Pneumonia in diseases classified elsewhere
J18.0	Bronchopneumonia, unspecified organism
J18.1	Lobar pneumonia, unspecified organism
J18.2	Hypostatic pneumonia, unspecified organism
J18.8	Other pneumonia, unspecified organism
J20.0	Acute bronchitis due to Mycoplasma pneumoniae
J20.1	Acute bronchitis due to Hemophilus influenzae
J20.2	Acute bronchitis due to streptococcus
J20.3	Acute bronchitis due to coxsackie virus
J20.4	Acute bronchitis due to parainfluenza virus
J20.5	Acute bronchitis due to respiratory syncytial virus
J20.6	Acute bronchitis due to rhinovirus
J20.7	Acute bronchitis due to echovirus
J20.8	Acute bronchitis due to other specified organisms
J20.9	Acute bronchitis, unspecified
J18.9	Pneumonia, unspecified organism
J21.0	Acute bronchiolitis due to respiratory syncytial virus
J21.1	Acute bronchiolitis due to human metapneumovirus
J21.8	Acute bronchiolitis due to other specified organisms
J21.9	Acute bronchiolitis, unspecified
J22.0	Unspecified acute lower respiratory infection

Data were examined by patients’ age, sex, hospital of admission, local health areas of residence, and admission date. Hospital length of stay (LOS) and accompanying diagnoses listed with the LRTI hospitalization were described. LOS was obtained directly from the DAD’s calculated LOS field, which is a derived variable. For inpatient abstracts, LOS is the difference in days between the admission date and discharge date. If the difference was 0 (i.e., admission date equals discharge date), the calculated LOS was 1. LOS was described for all pediatric patients, infants <1 year of age at the time of hospital admission, as well as for infants at high risk for an LRTI hospitalization. High-risk infants were defined according to the ICD-10 codes for congenital heart disease, bronchopulmonary dysplasia, congenital lung disease, and preterm birth defined as <37 weeks completed gestation [5,15,16] (Table 2).

**Table 2:** International Classification of Diseases, Tenth Revision (ICD-10) codes identifying high-risk conditions

**Condition**	**ICD-10 Code**	**Description**
Pre-term	P07.2	Extreme immaturity of newborn
	P07.3	Other preterm newborn
CHD	Q20	Congenital malformations of cardiac chambers and connections (select subcategories)
	Q20.0	Common arterial trunk
	Q20.1	Double outlet right ventricle
	Q20.2	Double outlet left ventricle
	Q20.3	Discordant ventriculoarterial connection
	Q20.4	Double inlet ventricle
	Q20.5	Discordant atrioventricular connection
	Q20.6	Isomerism of atrial appendages
	Q21	Congenital malformations of cardiac septa (select subcategories)
	Q21.0	Ventricular septal defect
	Q21.1	Atrial septal defect
	Q21.2	Atrioventricular septal defect
	Q21.3	Tetralogy of Fallot
	Q21.4	Aortopulmonary septal defect
	Q21.8	Other congenital malformations of cardiac septa
	Q22	Congenital malformations of pulmonary and tricuspid valves (all subcategories)
	Q23	Congenital malformations of aortic and mitral valves (all subcategories)
	Q24	Other congenital malformations of heart (all subcategories)
	Q25	Congenital malformations of great arteries (select subcategories)
	Q25.0	Patent ductus arteriosus
	Q25.1	Coarctation of aorta
	Q25.2	Atresia of aorta
	Q25.3	Stenosis of aorta
	Q25.4	Other congenital malformations of aorta
	Q25.5	Atresia of pulmonary artery
	Q25.6	Stenosis of pulmonary artery
	Q25.7	Other congenital malformations of pulmonary artery
	Q26	Congenital malformations of great veins (all subcategories)
BPD	P27.1	Bronchopulmonary dysplasia originating in the perinatal period
CLD	Q33	Congenital malformations of lung (all subcategories)

Average annual hospitalization rates for LRTI among the pediatric population were calculated. The overall hospitalization rates (per 100,000 children) were determined using the total number of LRTI hospitalizations and BC STATS PEOPLE 35 population estimates. Results are described as the number of hospital bed days used, and assuming a 100% utilization rate, results show the average number of beds required full time to care for LRTI patients during the study period of 2008 to 2010.

The projected increases in acute care beds and bed days for LRTI hospitalizations in 2020 and 2030 were calculated based on rates for 2008–2010 and population growth statistics provided by BC STATS. The best possible estimates of the effectiveness of public health initiatives to reduce the incidence of LRTI in 2020 and 2030 were estimated from a targeted literature review which analyzed the effects of non-pharmacological interventions on hospitalizations for severe viral respiratory disease. Based on this review, the most reasonable and appropriate benefit described as a decrease in the incidence of severe LRTI disease was applied to show the impact it would have on hospital days and beds needed in 2020 and 2030.

### Statistical analysis

All statistical analyses were performed with the use of MedCalc software, version 12.1.1 [17]. A MS Access 2007 database containing all inpatient records was used to store, manipulate, filter, and summarize the data. MS Excel 2007 was used for basic computations and to prepare charts.

Hospitalizations accounted for the total bed-days; the average number of beds was defined as total bed-days divided by the number of days in the period under analysis; average LOS was estimated as total bed-days divided by the number of hospitalizations; and the peak in bed occupancy was defined as the maximum number of beds occupied simultaneously.

Rates were calculated using the best population estimates available and the number of hospitalizations for corresponding groups defined by the patients’ primary diagnosis, age, and region of residence. The confidence intervals for these rates were calculated using the normal approximation method. Chi-square tests were used for rate comparisons, with Yates’ correction for continuity.

## Results

### All children (<19 years of age)

In British Columbia during 2008–2010, diseases of the respiratory system (ICD-10 Chapter X) accounted for 14326/186609 (7.7%) primary hospital diagnoses among all children <19 years of age. Hospitalizations for LRTI as the primary diagnosis occurred in 4588/14326 (32.0%) of all respiratory admissions in children <19 years of age. The average LOS in hospital for LRTI as the primary diagnosis was 3.10 days (95% confidence interval [CI] 3.03–3.21; 82% within 5 days); this is equivalent to a yearly average of 19.6 acute beds required to treat pediatric LRTIs (Table 3).

**Table 3:** Pediatric hospitalizations for lower respiratory tract infection as primary diagnosis, 2008–2010

	**Cases**	**Days**	**ALOS (95% CI)**	**Av Beds**	**Max. Beds**
All children	4588	14319	3.10 (3.03–3.21)	19.6	64
Infants <1y	2165	7002	3.23 (3.10–3.37)	9.6	41

Bed days are for separations within both fiscal years (length of stay of cases discharged).

Average beds calculated at 100% utilization (equivalent to average across year).

Maximum beds correspond to peak in daily bed utilization.

Most of the LRTI admissions were for an unspecified viral cause (2932/4588; 63.9%). Pneumonia, organism unspecified (ICD-10 code J18.9) was the most frequently listed LRTI as the primary diagnosis (1215/4588; 26.5%) with acute bronchiolitis due to RSV (ICD-10 code J21.0; 1198/4588; 26.1 %); acute bronchiolitis, unspecified (ICD-10 code J21.9; 854/4588; 18.6%); bronchopneumonia, unspecified organism (ICD-10 code J18.0; 354/4588; 7.7%); and viral pneumonia unspecified (ICD-10 code J12.9; 138/4588; 3%) also among the top 5 diagnoses.

RSV-specific infections accounted for 1353 (29.5%) cases of LRTI as the primary diagnosis among all children <19 years of age. Most RSV-associated hospitalizations were for acute bronchiolitis due to RSV accounting for 1198 (86%) RSV-associated hospitalizations, followed by RSV pneumonia (ICD-10 code J12.1; 115 cases; 8.5%), and acute bronchitis due to RSV (ICD-10 code J20.5; 40 cases; 3%).

### Infants (<1 year of age)

During 2008–2010, diseases of the respiratory system accounted for 2853/102972 (2.8%) primary diagnoses among infants <1 year of age (including deliveries/births). Hospitalizations for LRTI as the primary diagnosis occurred in 2165 (75.9%) infants <1 year of age. The average LOS for LRTI as the primary diagnosis was 3.23 days (95% CI 3.10–3.37; 79.5% within 5 days); this translates to a yearly average of 9.6 beds per day needed to treat LRTIs in infants <1 year of age (Table 3).

RSV-specific infections accounted for 1042 (48.1%) cases of LRTI as the primary diagnosis among infants <1 year of age. Most RSV-associated hospitalizations were for acute bronchiolitis due to RSV accounting for 961 (92.2%) RSV-associated hospitalizations, followed by RSV pneumonia (52 cases; 5%), and acute bronchitis due to RSV (29 cases; 2.8%).

Acute bronchiolitis due to RSV was the most frequently listed LRTI as the primary diagnosis (961/2165; 44.4%) followed by acute bronchiolitis, unspecified (575/2165; 26.6 %); pneumonia, unspecified organism (194/2165; 9%); bronchopneumonia, unspecified organism (102/2165; 4.7%); and acute bronchiolitis due to other specified organisms (ICD-10 code J21.8; 51/2165; 2.4%).

**Figure 1:**
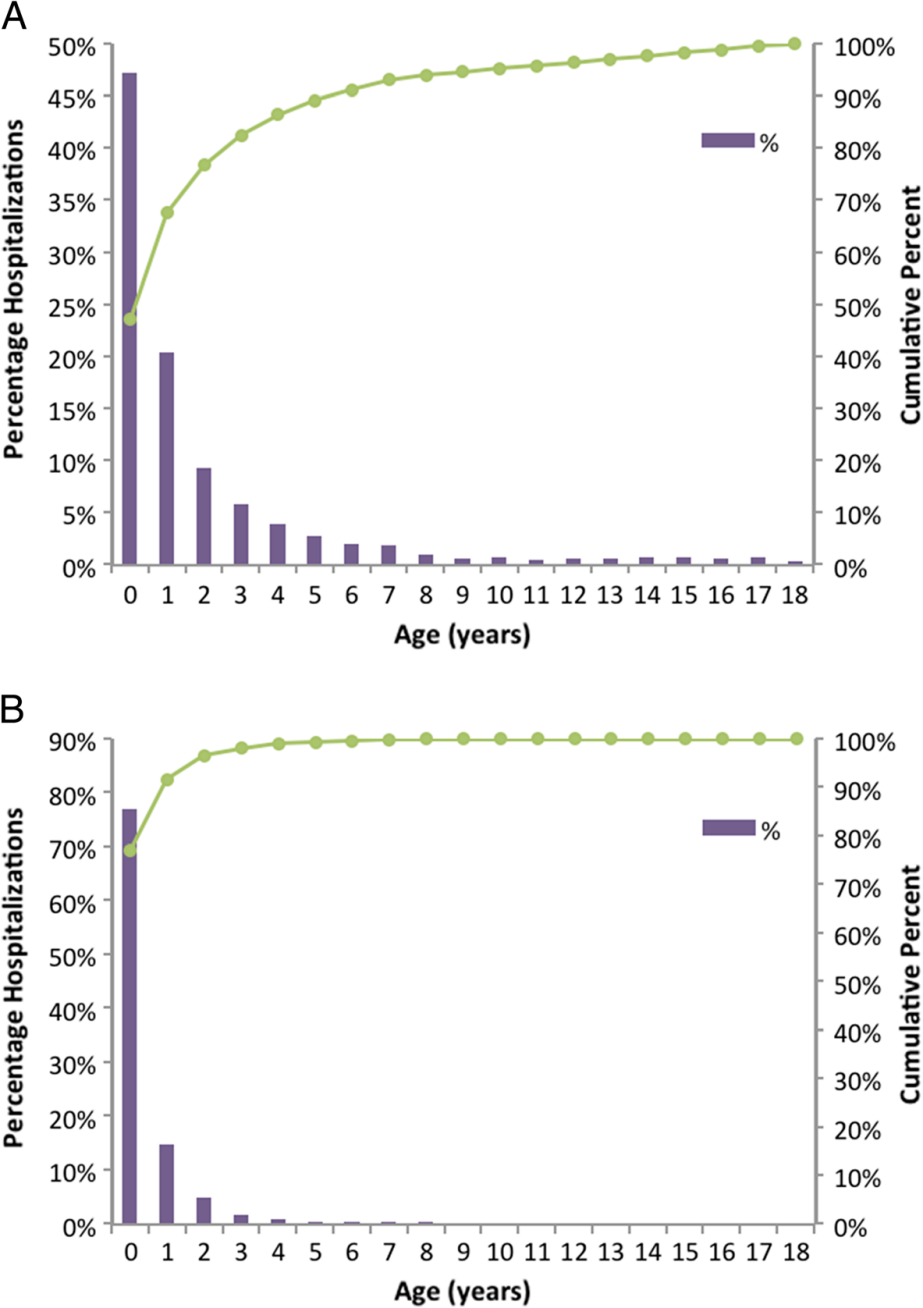
**Pediatric hospitalizations for LRTI and RSV according to age, 2008–1010. A**. Pediatric lower respiratory tract infection (LRTI) hospitalizations. Infants <1 year of age accounted for 47 % of LRTI hospitalizations among all children **B**. Pediatric respiratory syncytial virus (RSV) specific hospitalizations. Infants <1 year of age accounted for 77% RSV-specific LRTI hospitalizations among all children.

Infants <1 year of age accounted for 47 and 77% of LRTI and RSV-specific LRTI hospitalizations, respectively, among all children (Figure 1). Hospitalization rates for LRTI as the primary diagnosis among infants <1 year of age were significantly increased compared to children 1–<19 years of age (2410/100,000 population <1 year of age *vs*. 235/100,000 children 1–<19 years of age; P <0.0001). Bed-days rates for LRTI as the primary diagnosis were significantly increased among infants <1 year of age compared to children 1–<19 years of age (15,663/100,000 population <1year of age *vs*. 1473/100,000 children 1–<19 years of age; P <0.0001). Considering the bed days spent by those patients, this is an equivalent bed rate of 21.45 beds/100,000 infants <1 year of age per day and 2.02 beds/100,000 children 1–<19 years of age per day (P <0.0001).

### Infants at risk

At-risk infants accounted for 1.8% (40/2165) of LRTI hospitalizations in all infants <1 year of age. The average LOS among at-risk infants was significantly longer compared to other pediatric patients (9.1 days, *vs*. 3.10 days, respectively P <0.0001) (Table 4).

**Table 4:** Hospitalizations and average length of stay among high-risk compared to otherwise healthy patients, 2008–2010

	**Infants <1 y**		**Children 1–<19**		**Total**
**Risk level**	**Count**	**ALOS (95% CI)**	**Count**	**ALOS (95% CI)**	**Count**
High risk	40	9.10 (5.5–12.7)	38	4.3 (0–15.1)	78
Healthy	2125	3.10 (3.0–3.2)	2385	3.0 (2.9–3.1)	4510

ALOS, Average Length of Stay.

The average length of stay (ALOS) among at-risk infants was significantly longer compared to other pediatric patients (9.1 days, *vs*. 3.10 days, respectively P <0.0001).

### Seasonal and regional variations

**Figure 2:**
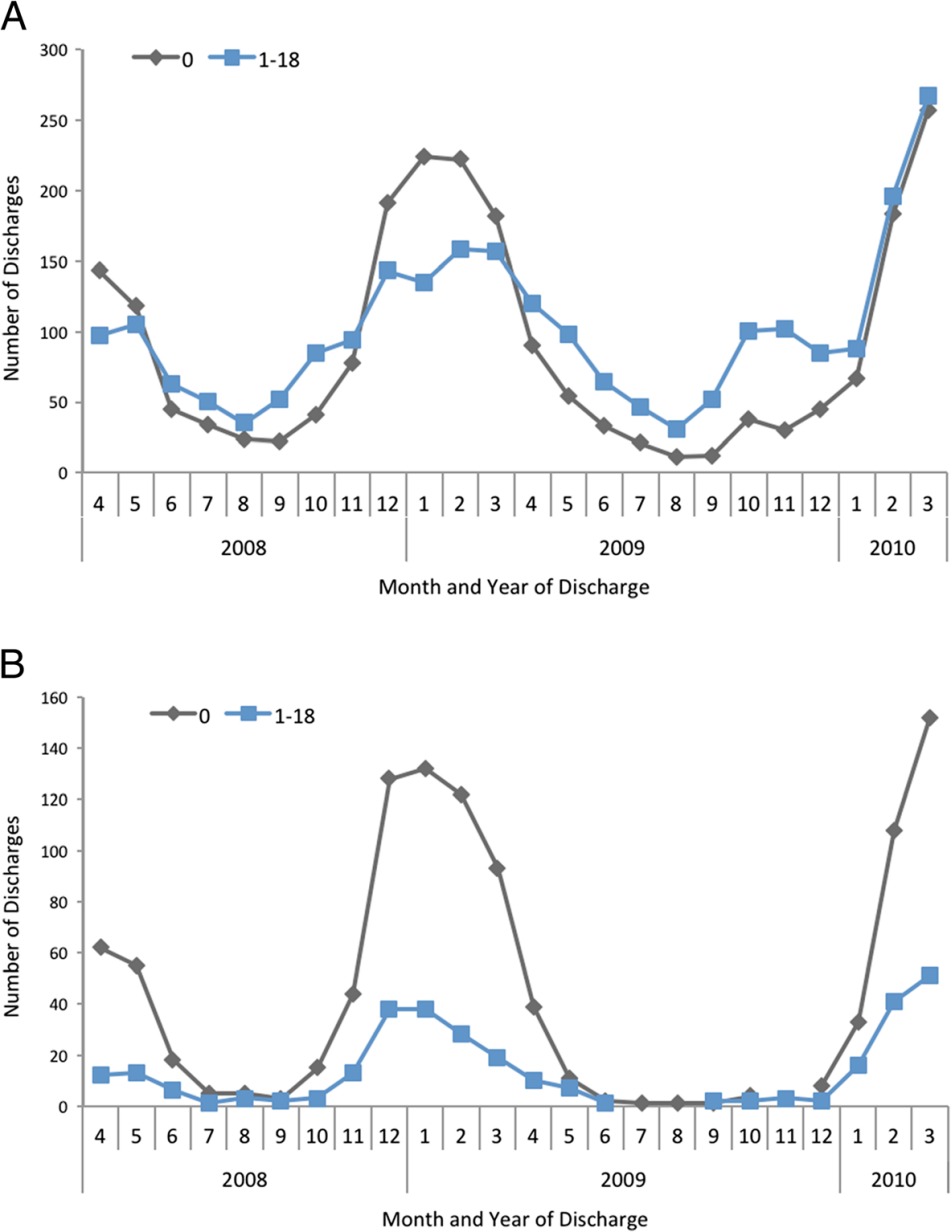
**Pediatric hospitalizations for LRTI and RSV according to month and year of discharge, 2008–2010. A**. Lower respiratory tract infection (LRTI) hospitalizations in all children. 73.1% LRTI hospitalizations among all children <19 years of age and 79.1% LRTI hospitalizations among infants <1 year of age occurred between November and April. **B**. Respiratory syncytial virus (RSV) specific hospitalizations in all children. 88.1% RSV hospitalizations among all children <19 years of age and 88.4% RSV hospitalizations among infants <1 year of age occurred between November and April.

Most LRTI hospitalizations among all children <19 years of age (LRTI, 73.1%; RSV, 88.1%) and infants <1 year of age (LRTI, 79.1%; RSV, 88.4%) occurred between November and April (Figure 2). Bed occupancy was highest in January and February of 2009 and March of 2010 with peaks of 53 beds among all children <19 years of age and 40 beds among infants <1 year of age in January and February 2009, respectively, and 64 beds among all children <19 years of age and 41 beds among infants <1 year of age in March 2010. Regionally, LRTI hospitalization rates among all children <19 years of age and infants <1 year of age in the Interior, Northern BC, and Vancouver Island were higher than the average provincial rate and significantly lower in British Columbia’s lower mainland (Table 5).

**Table 5:** Hospitalization rates for lower respiratory tract infection as primary diagnosis by region, 2008–10

**Health Authority**	**Infants**	**All children <19**
Interior	3049 (2634;3464) *	289 (263;316) *
Fraser	2030 (1821;2239) *	203 (188;217) *
Vancouver Coastal	1790 (1532;2047) *	174 (156;191) *
Vancouver Island	3085 (2661;3509) *	272 (246;299) *
Northern	3626 (3019;4234) *	373 (330;416) *
British Columbia	2410 (2309;2511)	235 (228;241)

Rates are per 100,000 population; 95% CI are shown in parentheses.

Rates for All Children include infants and patients up to 19 years of age.

(*) indicates significant difference (P value < 0.0001) in rates for health authority *vs*. provincial average.

Lower respiratory tract infection hospitalization rates among all children <19 years of age and infants <1 year of age in the Interior, Northern BC, and Vancouver Island were higher than the average provincial rate and significantly lower in British Columbia’s lower mainland.

### Projected LRTI hospitalizations for 2020 and 2030

Population projections estimated a 6.6% growth in the pediatric population by 2020 and 17.2% growth by 2030 (Table 6). Assuming the same incidence of LRTI hospitalizations required by pediatric patients, these projections predicted a growth in LRTI bed-days of 5.5% among all children <19 years of age and 16.3% among infants <1 year of age by 2020 and 16.2% among all children <19 years of age and 14.9% among infants <1 year of age by 2030. In 2020, the average number of acute care beds required to treat pediatric LRTIs will be 20.7 among all children 0–<19 years of age including 11.2 among the subpopulation of infants <1 year of age. This will increase to a maximum bed occupancy of 68 among all children including 48 in infants <1 year of age during the peak months for LRTI hospitalizations. In 2030, 22.8 acute care beds will be needed among all children <19 years of age including 11.0 among infants <1 year of age. This will increase to a maximum bed occupancy of 74 beds among all children including 47 among infants <1 year of age in the winter months (Table 7).

**Table 6:** British Columbia population estimates and projections to 2020 and 2030. Population projections estimated a 6.6% growth in the pediatric population by 2020 and 17.2% growth by 2030

**Age Group**	**2008**	**2020**	**2030**
Infants <1	44,156	52,032	51,410
All children <19	912,762	973,395	1,069,974

Source: BC Stats PEOPLE 35.

**Table 7:** Projections for lower respiratory tract infection hospitalizations with and without a public health intervention strategy

	**Year(s)**	**Hospitalizations**	**All Children**	**Bed-days**	**All Children**	**Beds**	**All Children**
		**Infants**		**Infants**		**Infants**	
Actual	2008-10	1,083	2,294	3,501	7,160	9.6 (41)	19.6 (64)
Proj1	2020	1,254	2,409	4,071	7,553	11.2 (48)	20.7 (68)
	2030	1,239	2,652	4,022	8,316	11.0 (47)	22.8 (74)
Proj2	2020	1,094	2,102	3,552	6,590	9.7 (41)	18.1 (59)
	2030	1,081	2,314	3,509	7,256	9.6 (41)	19.9 (65)

Beds correspond to annual average calculated from bed-days; peak in beds shown in parenthesis.

Proj1=projections based solely on population growth.

Proj2=Proj1 adjusted for public health intervention strategy.

Population projections predicted a growth in lower respiratory tract infection (LRTI) bed-days of 5.5% among all children <19 years of age and 16.3% among infants <1 year of age by 2020 and 16.2% among all children <19 years of age and 14.9% among infants <1 year of age by 2030. A public health prevention strategy that reduces LRTI hospitalizations by 12.5% may counteract the effects of population growth.

### Impact of public health prevention strategies on future LRTI hospitalizations

A targeted literature review quantitating the effect of non-pharmacological interventions on LRTI hospitalizations revealed a study that indicated a 12.5% reduction in hospitalizations may be achieved using hand-washing as a public health prevention strategy [18]. This reduction was applied to the projected increase in LRTI hospitalizations for 2020 and 2030 and indicated that a prevention strategy that could replicate these findings in reducing the prevalence of LRTI admissions, in a sense, counteracts the effects of population growth. In 2020, a 12.5% decrease in hospitalizations in an anticipated <19 year old British Columbia pediatric population of 1,025,000 could potentially result in 307 less hospitalizations, 963 less bed-days, and 2.6 less beds required annually to treat LRTIs. A 12.5% decrease in hospitalizations in an anticipated <1 year old British Columbia pediatric population of 52,032 could result in 160 less hospitalizations, 519 less bed-days, and 1.5 less beds. Similarly in 2030, in a projected <19 year old population of 1,128,585, there could be 338 less hospitalizations, 1060 less bed-days, and 2.9 less beds required annually to treat LRTIs. In a projected <1 year old population of 51,410, there could be 158 less hospitalizations, 513 less bed-days, and 1.4 less beds (Table 7).

## Discussion

LRTIs and particularly RSV associated LRTIs account for a substantial number of pediatric hospitalizations and utilize an important number of beds. In British Columbia during 2008–10, LRTI as the primary diagnosis accounted for a significant proportion of diseases of the respiratory system in all children <19 years of age and the majority of hospitalizations for diseases of the respiratory system in infants <1 year of age. Infants <1 year of age accounted for 47 and 77% hospitalizations due to pediatric LRTIs and LRTIs specifically due to RSV, respectively. Acute bronchiolitis due to RSV was the most frequently listed LRTI as the primary diagnosis in infants <1 year of age. There was considerable seasonal variation in the incidence of hospitalizations. Most pediatric hospitalizations attributable to LRTIs in British Columbia hospitals occurred between November and April.

These data demonstrate that LRTI and RSV specific LRTI are a significant disease burden in the pediatric population in British Columbia, especially in infants <1 year of age and those at high-risk. Although high-risk infants do not account for the majority of the number of pediatric LRTI hospitalizations, their hospital LOS was three times as long as other pediatric patients. This suggests at-risk infants place a greater demand per patient on the acute care setting. Previous studies show the lengths of RSV-associated stay in Canada range from 4.6 to 6.7 days for pediatric patients, and from 8.6 to 11.8 days for pediatric patients with diagnoses such as congenital heart disease, chronic lung disease, immunodeficiency, or multiple congenital anomalies, [19] and up to 14.71 days for preterm infants with probable RSV compared to 5.04 days for term infants [20]. These data are in accordance with our observations in British Columbia.

Due to the high rate of pediatric hospitalization and extended lengths of stay, there are significant medical costs related to LRTI. Previous economic analyses indicate that infants with LRTI have healthcare costs over $9000 higher in the first year of life than infants without infection [21]. The LRTI burden on the healthcare system in British Columbia is expected to increase in the next 20 years as estimates of population growth suggest that the number of acute care beds needed to care for pediatric LRTI hospitalizations will rise. The study estimated an increase in the pediatric population of approximately 17% by 2030 which may be a conservative estimate compared to other published reports which calculate 31% growth in the population of children 0–19 years of age in British Columbia from 2010 to 2031 [22]. However, predictions describing the implementation and evaluation of effective public health prevention strategies indicate that such measures can potentially reduce the incidence of the severe viral infections that are predominant contributors to LRTI hospitalizations and can significantly contribute to pediatric acute care sustainability. Non-pharmacological intervention strategies can occur in the home, and health care practitioners need to educate parents and caregivers about methods that can be used to prevent children from infections. These include frequent hand washing which can significantly reduce the rate of spread of infection from 4.2 to 0.6–1.1% [23] and separating infected people from non-infected people, especially at-risk children [24]. The effectiveness of other public health initiatives warrants evaluation.

The significance of developing public health response strategies to infectious diseases was illustrated during the 2009 H1N1 influenza pandemic. Public opinion polls demonstrated that 59–67% Americans adopted hand washing and 35–38% avoided exposure to others with influenza-like symptoms [10]. While the effectiveness of such awareness campaigns are difficult to ascertain, strategies that can decrease the prevalence of LRTIs may have considerable impact on acute care settings especially during seasonal LRTI and RSV outbreaks. Compliance with vaccines that are efficacious at reducing the incidence of severe viral respiratory infections [25] or the use of monoclonal antibodies to reduce the risk of RSV hospitalization in high-risk infants [26] may also have a significant contribution to reducing the demand of LRTIs on pediatric inpatient settings. In some areas, a modified combination of prevention may be useful. This may be applicable in British Columbia in the Interior, Northern BC, and Vancouver Island where LRTI hospitalization rates among all children <19 years of age and infants <1 year of age were higher than the average provincial rate. Furthermore, this study revealed that the primary diagnosis for LRTIs were predominantly for an unspecified cause. Improved surveillance on the cause of pediatric LRTI hospitalizations may provide valuable insight for assessing current and future demand as well as for developing targeted prevention strategies.

Our study reflects the impact of LRTIs in a closed population [14]. This may be an advantage over other nationwide administrative analyses that only evaluate a sample of a population. There are however limitations associated with this study. First, the use of ICD-10 codes with hospital discharge diagnoses to estimate LRTI hospitalizations may be unsatisfactory as discharge diagnoses can be miscoded. Second, as previously noted the sensitivity of the use of LRTI ICD-10 codes is uncertain as most of the LRTI hospitalizations were for an unspecified viral cause. The study was unable to determine if each hospitalization had a laboratory investigation to determine the underlying cause. Third, it is difficult to evaluate the impact of health prevention strategies in decreasing severe respiratory disease as the effectiveness of such measures may only be quantified using the best estimates available in the literature. Fourth, the future predictions on demand were based on 2008–2010 data only, whereby the retrospective inclusion of more years would potentially improve the accuracy of estimating the temporal trend and demand. Fifth, the study only assessed the inpatient utilization of pediatrics with a primary LRTI diagnosis, whereby those with secondary diagnoses, for example those acquiring nosocomial LRTI, were not included. Finally, while our findings suggest that LRTIs are a significant burden on the healthcare system in British Columbia and public health prevention strategies can severely impact this, the generalizability of this data to other provinces in Canada and other countries is unknown.

## Conclusion

In summary, we present data for all pediatric LRTI and RSV/LRTI hospitalization occurrences in British Columbia during 2008–2010. We identified pediatric LRTIs, and specifically in infants <1 year of age and at-risk infants, as a significant burden on the acute care setting in British Columbia, having pronounced seasonal variability. Population projections suggest that this burden will increase in the next 20 years whereby the implementation of effective public health prevention strategies may be an important strategy for pediatric healthcare sustainability, particularly as it relates to acute care access with fixed infrastructure. Information about the risk of hospitalization in the pediatric population along with a greater understanding of the factors involved in the seasonality patterns of LRTI hospitalizations and the etiology for the LRTI hospitalizations may contribute to the development of algorithms that can identify risk and allow the development of prevention strategies to be implemented at specific times of the year. Comprehensive knowledge of the province-specific impact and cause of pediatric LRTIs can lead to the development of prevention strategies that if effectively implemented can ease the burden of disease on patients, health care infrastructure, and budgets, all of which are important for healthcare sustainability. The methodology and analyses used to evaluate the burden of pediatric LRTIs in British Columbia may serve as a model that can be extrapolated for use in other Canadian provinces and countries.

## Abbreviations

LRTI: Lower Respiratory Tract Infection; RSV: Respiratory Syncytial Virus.

## Competing interests

YS has no competing interests or conflicts of interest to disclose.

## Authors’ contributions

PS participated in the study design, identified the data requirements, carried out the analysis and helped to draft the manuscript. KG conceived of the study, participated in its design and coordination, provided interpretation to the results and drafted the manuscript. PV participated in the interpretation of results. ML participated in the design and coordination of the study and drafting of manuscript. YS arranged the provision of data and contributed in the interpretation of the analysis. All authors read and approved the final manuscript.

## Ethics statement

Non-identifiable administrative data was utilized for the purposes of this analysis and therefore ethics approval was not required.

## Pre-publication history

The pre-publication history for this paper can be accessed here:

http://www.biomedcentral.com/1472-6963/12/451/prepub
